# (2*E*)-3-(2-Chloro-8-methyl­quinolin-3-yl)-1-(2,4-di­methyl­quinolin-3-yl)prop-2-en-1-one

**DOI:** 10.1107/S1600536813019405

**Published:** 2013-07-20

**Authors:** R. Prasath, S. Sarveswari, Seik Weng Ng, Edward R. T. Tiekink

**Affiliations:** a Department of Chemistry, BITS, Pilani - K. K. Birla Goa Campus, Goa 403 726, India; b Organic Chemistry Division, School of Advanced Sciences, VIT University, Vellore 632 014, India; c Department of Chemistry, University of Malaya, 50603 Kuala Lumpur, Malaysia; d Chemistry Department, Faculty of Science, King Abdulaziz University, PO Box 80203 Jeddah, Saudi Arabia

## Abstract

In the mol­ecule of the title compound, C_24_H_19_ClN_2_O, the terminal quinolinyl residues are close to perpendicular to each other [dihedral angle 83.72 (4)°]. The quinolinyl residues are connected by and inclined to the prop-2-en-1-one bridge, with the C_ar_—C_ar_—C—C (ar = aromatic) torsion angles being 71.01 (17) and 20.6 (2)°. The crystal structure features phen­yl–carbonyl C—H⋯O inter­actions and π–π inter­actions between centrosymmetrically related quinolinyl residues [3.5341 (10) and 3.8719 (9) Å], which together lead to a three-dimensional architecture.

## Related literature

For background to quinoline chalcones and for a related structure, see: Prasath *et al.* (2013 ▶).
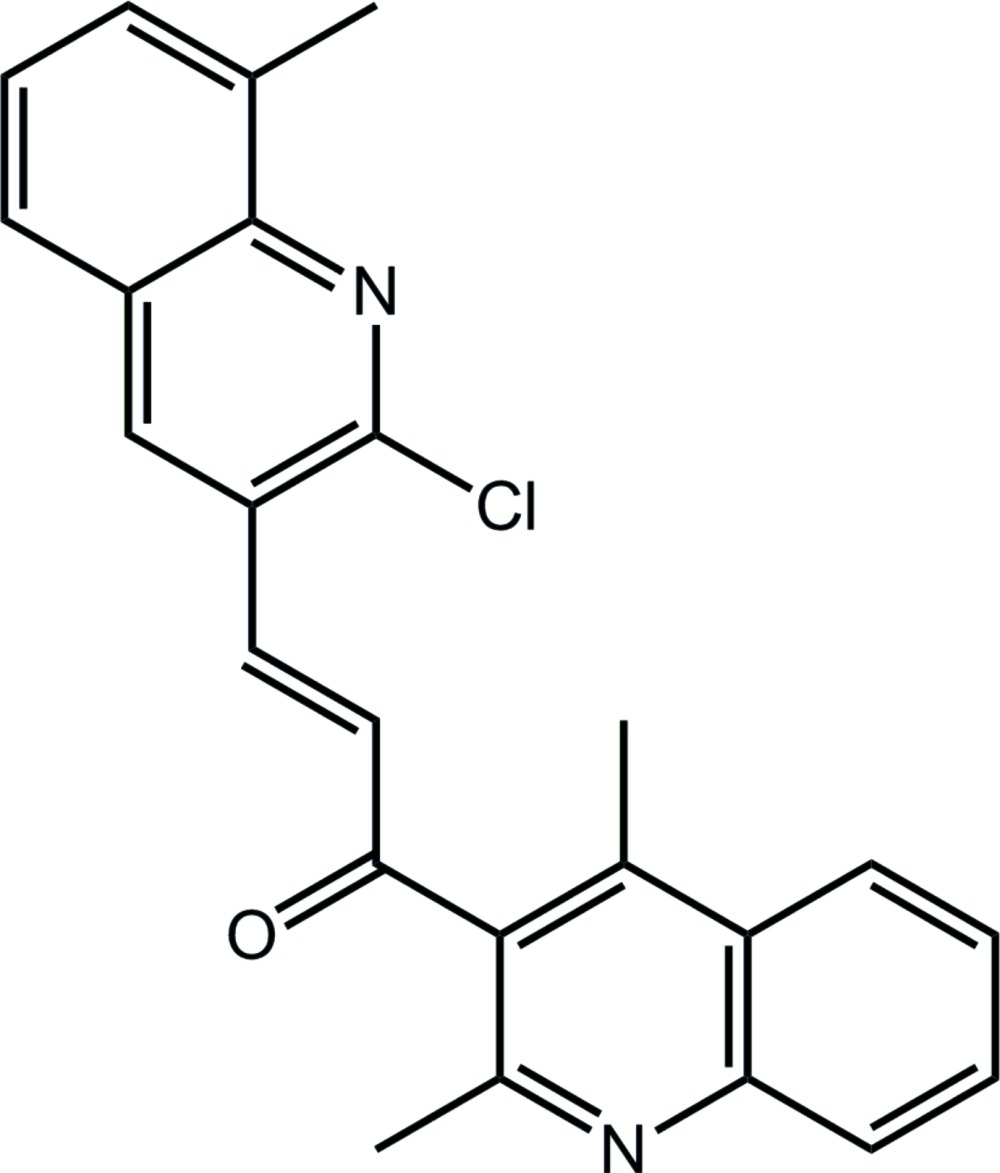


## Experimental

### 

#### Crystal data

C_24_H_19_ClN_2_O
*M* *_r_* = 386.86
Triclinic, 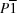

*a* = 7.4150 (5) Å
*b* = 9.9045 (6) Å
*c* = 14.0696 (9) Å
α = 71.072 (5)°
β = 88.427 (5)°
γ = 72.552 (5)°
*V* = 929.66 (10) Å^3^
*Z* = 2
Cu *K*α radiation
μ = 1.95 mm^−1^
*T* = 100 K
0.25 × 0.25 × 0.25 mm

#### Data collection

Agilent SuperNova Dual diffractometer with Atlas detector
Absorption correction: multi-scan (*CrysAlis PRO*; Agilent, 2013 ▶) *T*_min_ = 0.853, *T*_max_ = 1.000
6846 measured reflections
3811 independent reflections
3587 reflections with *I* > 2σ(*I*)
*R*_int_ = 0.014

#### Refinement

*R*[*F* ^2^ > 2σ(*F* ^2^)] = 0.035
*wR*(*F* ^2^) = 0.095
*S* = 1.04
3811 reflections
256 parameters
H-atom parameters constrained
Δρ_max_ = 0.30 e Å^−3^
Δρ_min_ = −0.30 e Å^−3^

### 

Data collection: *CrysAlis PRO* (Agilent, 2013 ▶); cell refinement: *CrysAlis PRO*; data reduction: *CrysAlis PRO*; program(s) used to solve structure: *SHELXS97* (Sheldrick, 2008 ▶); program(s) used to refine structure: *SHELXL97* (Sheldrick, 2008 ▶); molecular graphics: *ORTEP-3 for Windows* (Farrugia, 2012 ▶) and *DIAMOND* (Brandenburg, 2006 ▶); software used to prepare material for publication: *publCIF* (Westrip, 2010 ▶).

## Supplementary Material

Crystal structure: contains datablock(s) global, I. DOI: 10.1107/S1600536813019405/hb7108sup1.cif

Structure factors: contains datablock(s) I. DOI: 10.1107/S1600536813019405/hb7108Isup2.hkl

Supplementary material file. DOI: 10.1107/S1600536813019405/hb7108Isup3.cml

Additional supplementary materials:  crystallographic information; 3D view; checkCIF report

## Figures and Tables

**Table 1:** Hydrogen-bond geometry (Å, °)

*D*—H⋯*A*	*D*—H	H⋯*A*	*D*⋯*A*	*D*—H⋯*A*
C20—H20⋯O1^i^	0.95	2.58	3.364 (2)	140

Symmetry code: (i) 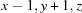.

**Fig. 1.**
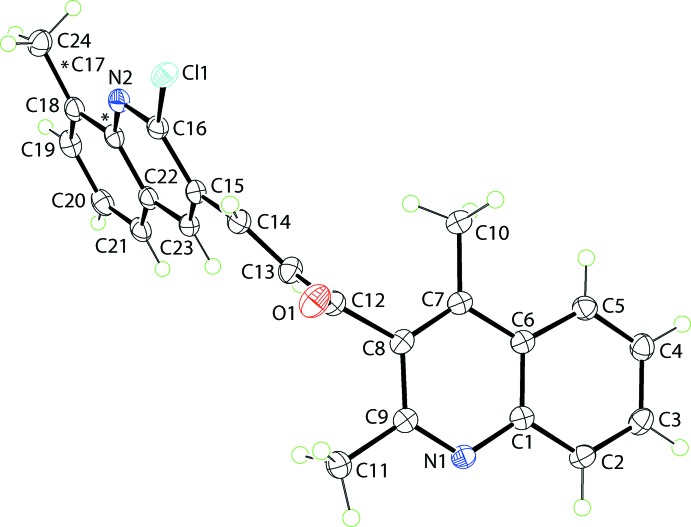
The molecular structure of (I), showing displacement ellipsoids at the 50% probability level.

**Fig. 2.**
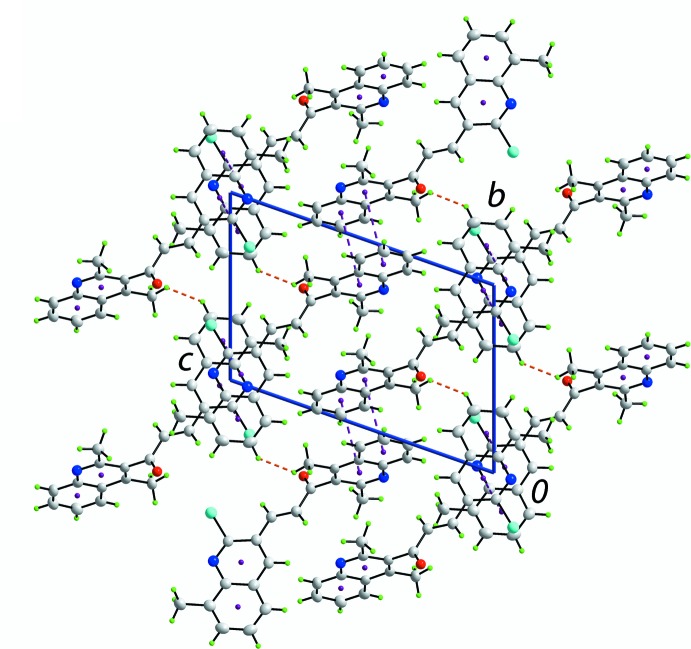
A view, in projection down the a axis, of the unit-cell contents of (I). The C—H···O and π—π interactions are shown as orange and purple blue dashed lines, respectively.

## supplementary crystallographic information

### Comment

For the background to biological activities, utility as intermediates in organic synthesis and photophysical properties of quinolines, as well as the bio-activities of quinolinyl chalcones and a related structure, see the Introduction to Prasath *et al.* (2013).

In (I), Fig. 1, the dihedral angle between the quinolinyl rings is 83.72 (4)°. The conformation about the ethylene bond [C13═C14 = 1.3333 (19) Å] is *E*. The central prop-2-en-1-one residue, comprising the O1 and C12–C15 atoms, is twisted, as manifested in the O1—C12—C13—C14 torsion angle of 16.4 (2)°. The N1- and N2-containing quinolinyl rings are also twisted with respect to the central bridge, as seen in the C7—C8—C12—C13 and C13—C14—C15—C23 torsion angles of 71.01 (17) and 20.6 (2)°, respectively.

In the most closely related structure available for comparison, (II), namely (2*E*)-3-(6-chloro-2-methoxyquinolin-3-yl)-1-(5,7-dimethylquinolin-6-yl)prop-2-en-1-one (Prasath *et al.*, 2013), the dihedral angle between the quinolinyl residues is 63.30 (5)°, indicating a more compact configuration than that in (I); the central residue in (II) is planar. Finally, when the structures are viewed normal to the ethylene bond, the pyridyl-N atoms in (I) can be described as *anti*, whereas they are closer to *syn* in (II).

In the crystal, linear supramolecular chains are formed by phenyl-C—H···O(carbonyl) interactions, Table 1. These, along with π–π interactions between the rings of centrosymmetrically related N1-quinolinyl residues [3.7578 (9) Å; angle of inclination = 1.91 (7)° for symmetry operation 1 - *x*, -*y*, 1 - *z*] and between the rings of centrosymmetrically related N2-quinolinyl residues [3.5767 (9) Å; angle of inclination = 0.99 (7)° for symmetry operation -*x*, 2 - *y*, -*z*], connect the molecules into a three-dimensional architecture, Fig. 2.

### Experimental

A mixture of 2,4-dimethyl-3-acetylquinoline (200 mg, 0.001 *M*) and 2-chloro-8-methylquinoline-3-carbaldehyde (200 mg, 0.001 *M*) in methanol (20 ml) containing 0.2 g of potassium hydroxide was stirred at room temperature for 12 h. Then the reaction mixture was neutralized with dilute acetic acid and the resultant solid was filtered, dried and purified by column chromatography using ethyl acetate–hexane (1:1) mixture to afford (I). Re-crystallization was by slow evaporation of an acetone solution of (I), which yielded pale-yellow blocks in 78% yield; m.p. 458–460 K.

### Refinement

Carbon-bound H atoms were placed in calculated positions [C—H = 0.95–0.98 Å, *U*_iso_(H) = 1.2–1.5*U*_eq_(C)] and were included in the refinement in the riding-model approximation.

### Crystal data

**Table:**

C_24_H_19_ClN_2_O	*Z* = 2
*M**_r_* = 386.86	*F*(000) = 404
Triclinic, *P*1	*D*_x_ = 1.382 Mg m^−^^3^
Hall symbol: -P 1	Cu *K*α radiation, λ = 1.54184 Å
*a* = 7.4150 (5) Å	Cell parameters from 4165 reflections
*b* = 9.9045 (6) Å	θ = 3.3–76.5°
*c* = 14.0696 (9) Å	µ = 1.95 mm^−^^1^
α = 71.072 (5)°	*T* = 100 K
β = 88.427 (5)°	Block, pale-yellow
γ = 72.552 (5)°	0.25 × 0.25 × 0.25 mm
*V* = 929.66 (10) Å^3^	

### Data collection

**Table:**

Agilent SuperNova Dual diffractometer with Atlas detector	3811 independent reflections
Radiation source: SuperNova (Cu) X-ray Source	3587 reflections with *I* > 2σ(*I*)
Mirror monochromator	*R*_int_ = 0.014
Detector resolution: 10.4041 pixels mm^-1^	θ_max_ = 76.7°, θ_min_ = 3.3°
ω scans	*h* = −9→6
Absorption correction: multi-scan (*CrysAlis PRO*; Agilent, 2013)	*k* = −12→11
*T*_min_ = 0.853, *T*_max_ = 1.000	*l* = −17→17
6846 measured reflections	

### Refinement

**Table:**

Refinement on *F*^2^	Primary atom site location: structure-invariant direct methods
Least-squares matrix: full	Secondary atom site location: difference Fourier map
*R*[*F*^2^ > 2σ(*F*^2^)] = 0.035	Hydrogen site location: inferred from neighbouring sites
*wR*(*F*^2^) = 0.095	H-atom parameters constrained
*S* = 1.04	*w* = 1/[σ^2^(*F*_o_^2^) + (0.0508*P*)^2^ + 0.4107*P*] where *P* = (*F*_o_^2^ + 2*F*_c_^2^)/3
3811 reflections	(Δ/σ)_max_ < 0.001
256 parameters	Δρ_max_ = 0.30 e Å^−^^3^
0 restraints	Δρ_min_ = −0.30 e Å^−^^3^

### Special details

**Table:**

Geometry. All s.u.'s (except the s.u. in the dihedral angle between two l.s. planes) are estimated using the full covariance matrix. The cell s.u.'s are taken into account individually in the estimation of s.u.'s in distances, angles and torsion angles; correlations between s.u.'s in cell parameters are only used when they are defined by crystal symmetry. An approximate (isotropic) treatment of cell s.u.'s is used for estimating s.u.'s involving l.s. planes.
Refinement. Refinement of *F*^2^ against ALL reflections. The weighted *R*-factor *wR* and goodness of fit *S* are based on *F*^2^, conventional *R*-factors *R* are based on *F*, with *F* set to zero for negative *F*^2^. The threshold expression of *F*^2^ > 2σ(*F*^2^) is used only for calculating *R*-factors(gt) *etc*. and is not relevant to the choice of reflections for refinement. *R*-factors based on *F*^2^ are statistically about twice as large as those based on *F*, and *R*-factors based on ALL data will be even larger.

### Fractional atomic coordinates and isotropic or equivalent isotropic displacement parameters (Å^2^)

**Table:**

	*x*	*y*	*z*	*U*_iso_*/*U*_eq_	
Cl1	0.50310 (4)	0.74397 (4)	−0.07296 (2)	0.02306 (10)	
O1	0.77211 (15)	0.37500 (12)	0.27778 (8)	0.0303 (2)	
N1	0.59497 (15)	0.23114 (12)	0.58173 (8)	0.0197 (2)	
N2	0.27853 (15)	0.99608 (13)	−0.06433 (8)	0.0204 (2)	
C1	0.45484 (18)	0.16757 (15)	0.57864 (10)	0.0191 (3)	
C2	0.40578 (19)	0.08004 (16)	0.67102 (10)	0.0230 (3)	
H2	0.4675	0.0690	0.7326	0.028*	
C3	0.2705 (2)	0.01120 (16)	0.67258 (11)	0.0248 (3)	
H3	0.2387	−0.0471	0.7351	0.030*	
C4	0.1783 (2)	0.02640 (16)	0.58199 (11)	0.0250 (3)	
H4	0.0863	−0.0233	0.5835	0.030*	
C5	0.22039 (19)	0.11248 (15)	0.49156 (10)	0.0222 (3)	
H5	0.1563	0.1227	0.4309	0.027*	
C6	0.35811 (18)	0.18627 (14)	0.48764 (10)	0.0187 (3)	
C7	0.40656 (18)	0.27748 (14)	0.39547 (10)	0.0191 (3)	
C8	0.55043 (18)	0.33647 (14)	0.40032 (10)	0.0192 (3)	
C9	0.64203 (18)	0.31126 (14)	0.49597 (10)	0.0189 (3)	
C10	0.3031 (2)	0.30187 (16)	0.29759 (10)	0.0234 (3)	
H10A	0.3453	0.3727	0.2423	0.035*	
H10B	0.1664	0.3426	0.3016	0.035*	
H10C	0.3304	0.2061	0.2853	0.035*	
C11	0.79125 (19)	0.38402 (16)	0.50126 (11)	0.0232 (3)	
H11A	0.8428	0.3514	0.5713	0.035*	
H11B	0.7346	0.4933	0.4766	0.035*	
H11C	0.8934	0.3547	0.4594	0.035*	
C12	0.61717 (19)	0.42581 (16)	0.30561 (10)	0.0219 (3)	
C13	0.4853 (2)	0.57651 (16)	0.25090 (10)	0.0233 (3)	
H13	0.3830	0.6205	0.2844	0.028*	
C14	0.50609 (19)	0.65201 (16)	0.15594 (10)	0.0216 (3)	
H14	0.6057	0.6040	0.1228	0.026*	
C15	0.38631 (18)	0.80355 (15)	0.09910 (10)	0.0204 (3)	
C16	0.37602 (18)	0.86237 (15)	−0.00807 (10)	0.0198 (3)	
C17	0.17413 (18)	1.09272 (15)	−0.01732 (10)	0.0203 (3)	
C18	0.06749 (19)	1.24027 (16)	−0.07813 (11)	0.0226 (3)	
C19	−0.0346 (2)	1.33729 (16)	−0.03049 (12)	0.0261 (3)	
H19	−0.1056	1.4363	−0.0702	0.031*	
C20	−0.0373 (2)	1.29435 (17)	0.07542 (12)	0.0264 (3)	
H20	−0.1087	1.3644	0.1057	0.032*	
C21	0.0626 (2)	1.15243 (17)	0.13450 (11)	0.0243 (3)	
H21	0.0597	1.1235	0.2057	0.029*	
C22	0.17080 (19)	1.04805 (16)	0.08914 (10)	0.0209 (3)	
C23	0.27938 (19)	0.90076 (16)	0.14569 (10)	0.0213 (3)	
H23	0.2790	0.8678	0.2171	0.026*	
C24	0.0660 (2)	1.28611 (17)	−0.19129 (11)	0.0274 (3)	
H24A	−0.0111	1.3913	−0.2211	0.041*	
H24B	0.0120	1.2227	−0.2151	0.041*	
H24C	0.1959	1.2741	−0.2113	0.041*	

### Atomic displacement parameters (Å^2^)

**Table:**

	*U*^11^	*U*^22^	*U*^33^	*U*^12^	*U*^13^	*U*^23^
Cl1	0.02514 (17)	0.02405 (17)	0.01915 (17)	−0.00578 (13)	0.00524 (12)	−0.00798 (12)
O1	0.0269 (5)	0.0329 (6)	0.0264 (5)	−0.0062 (4)	0.0094 (4)	−0.0069 (4)
N1	0.0197 (5)	0.0203 (5)	0.0189 (5)	−0.0053 (4)	0.0017 (4)	−0.0067 (4)
N2	0.0193 (5)	0.0222 (6)	0.0213 (5)	−0.0090 (4)	0.0033 (4)	−0.0071 (5)
C1	0.0180 (6)	0.0184 (6)	0.0198 (6)	−0.0032 (5)	0.0017 (5)	−0.0072 (5)
C2	0.0235 (6)	0.0255 (7)	0.0181 (6)	−0.0068 (5)	0.0009 (5)	−0.0053 (5)
C3	0.0241 (7)	0.0251 (7)	0.0213 (7)	−0.0078 (6)	0.0041 (5)	−0.0028 (5)
C4	0.0221 (6)	0.0243 (7)	0.0276 (7)	−0.0091 (5)	0.0018 (5)	−0.0055 (6)
C5	0.0209 (6)	0.0218 (6)	0.0230 (7)	−0.0056 (5)	−0.0006 (5)	−0.0069 (5)
C6	0.0181 (6)	0.0175 (6)	0.0190 (6)	−0.0023 (5)	0.0017 (5)	−0.0069 (5)
C7	0.0195 (6)	0.0176 (6)	0.0180 (6)	−0.0015 (5)	0.0011 (5)	−0.0067 (5)
C8	0.0200 (6)	0.0174 (6)	0.0182 (6)	−0.0029 (5)	0.0038 (5)	−0.0059 (5)
C9	0.0181 (6)	0.0177 (6)	0.0196 (6)	−0.0026 (5)	0.0026 (5)	−0.0073 (5)
C10	0.0255 (7)	0.0241 (7)	0.0189 (6)	−0.0072 (5)	−0.0004 (5)	−0.0052 (5)
C11	0.0212 (6)	0.0235 (7)	0.0256 (7)	−0.0079 (5)	0.0027 (5)	−0.0083 (5)
C12	0.0236 (6)	0.0238 (7)	0.0195 (6)	−0.0092 (5)	0.0037 (5)	−0.0073 (5)
C13	0.0255 (7)	0.0225 (7)	0.0218 (6)	−0.0082 (5)	0.0052 (5)	−0.0065 (5)
C14	0.0221 (6)	0.0239 (7)	0.0208 (6)	−0.0093 (5)	0.0041 (5)	−0.0080 (5)
C15	0.0198 (6)	0.0224 (7)	0.0210 (6)	−0.0109 (5)	0.0031 (5)	−0.0061 (5)
C16	0.0191 (6)	0.0225 (6)	0.0207 (6)	−0.0096 (5)	0.0042 (5)	−0.0083 (5)
C17	0.0174 (6)	0.0220 (7)	0.0243 (7)	−0.0101 (5)	0.0033 (5)	−0.0079 (5)
C18	0.0200 (6)	0.0233 (7)	0.0260 (7)	−0.0107 (5)	0.0023 (5)	−0.0066 (5)
C19	0.0212 (6)	0.0216 (7)	0.0355 (8)	−0.0074 (5)	0.0018 (6)	−0.0087 (6)
C20	0.0222 (6)	0.0275 (7)	0.0362 (8)	−0.0107 (6)	0.0080 (6)	−0.0172 (6)
C21	0.0235 (7)	0.0296 (7)	0.0274 (7)	−0.0150 (6)	0.0076 (5)	−0.0134 (6)
C22	0.0196 (6)	0.0244 (7)	0.0239 (7)	−0.0126 (5)	0.0044 (5)	−0.0096 (5)
C23	0.0225 (6)	0.0252 (7)	0.0199 (6)	−0.0134 (5)	0.0039 (5)	−0.0072 (5)
C24	0.0267 (7)	0.0250 (7)	0.0262 (7)	−0.0073 (6)	0.0006 (6)	−0.0036 (6)

### Geometric parameters (Å, º)

**Table:**

Cl1—C16	1.7554 (14)	C11—H11A	0.9800
O1—C12	1.2177 (17)	C11—H11B	0.9800
N1—C9	1.3150 (17)	C11—H11C	0.9800
N1—C1	1.3749 (17)	C12—C13	1.4825 (19)
N2—C16	1.2963 (18)	C13—C14	1.3333 (19)
N2—C17	1.3752 (18)	C13—H13	0.9500
C1—C6	1.4161 (18)	C14—C15	1.4636 (19)
C1—C2	1.4161 (18)	C14—H14	0.9500
C2—C3	1.368 (2)	C15—C23	1.3814 (19)
C2—H2	0.9500	C15—C16	1.4243 (18)
C3—C4	1.406 (2)	C17—C22	1.4193 (19)
C3—H3	0.9500	C17—C18	1.423 (2)
C4—C5	1.370 (2)	C18—C19	1.378 (2)
C4—H4	0.9500	C18—C24	1.507 (2)
C5—C6	1.4136 (19)	C19—C20	1.412 (2)
C5—H5	0.9500	C19—H19	0.9500
C6—C7	1.4269 (18)	C20—C21	1.366 (2)
C7—C8	1.3739 (19)	C20—H20	0.9500
C7—C10	1.5082 (18)	C21—C22	1.4221 (19)
C8—C9	1.4371 (18)	C21—H21	0.9500
C8—C12	1.5050 (18)	C22—C23	1.410 (2)
C9—C11	1.5055 (18)	C23—H23	0.9500
C10—H10A	0.9800	C24—H24A	0.9800
C10—H10B	0.9800	C24—H24B	0.9800
C10—H10C	0.9800	C24—H24C	0.9800
			
C9—N1—C1	118.17 (11)	O1—C12—C8	120.60 (12)
C16—N2—C17	117.78 (12)	C13—C12—C8	116.16 (11)
N1—C1—C6	122.93 (12)	C14—C13—C12	121.82 (13)
N1—C1—C2	118.08 (12)	C14—C13—H13	119.1
C6—C1—C2	119.00 (12)	C12—C13—H13	119.1
C3—C2—C1	120.74 (13)	C13—C14—C15	124.62 (13)
C3—C2—H2	119.6	C13—C14—H14	117.7
C1—C2—H2	119.6	C15—C14—H14	117.7
C2—C3—C4	120.19 (13)	C23—C15—C16	115.37 (12)
C2—C3—H3	119.9	C23—C15—C14	122.38 (12)
C4—C3—H3	119.9	C16—C15—C14	122.23 (12)
C5—C4—C3	120.42 (13)	N2—C16—C15	126.49 (13)
C5—C4—H4	119.8	N2—C16—Cl1	115.42 (10)
C3—C4—H4	119.8	C15—C16—Cl1	118.08 (10)
C4—C5—C6	120.68 (13)	N2—C17—C22	121.50 (13)
C4—C5—H5	119.7	N2—C17—C18	118.38 (12)
C6—C5—H5	119.7	C22—C17—C18	120.12 (13)
C5—C6—C1	118.94 (12)	C19—C18—C17	118.10 (13)
C5—C6—C7	122.83 (12)	C19—C18—C24	121.77 (13)
C1—C6—C7	118.22 (12)	C17—C18—C24	120.12 (13)
C8—C7—C6	117.71 (12)	C18—C19—C20	122.18 (14)
C8—C7—C10	122.91 (12)	C18—C19—H19	118.9
C6—C7—C10	119.36 (12)	C20—C19—H19	118.9
C7—C8—C9	120.42 (12)	C21—C20—C19	120.24 (13)
C7—C8—C12	120.60 (12)	C21—C20—H20	119.9
C9—C8—C12	118.97 (12)	C19—C20—H20	119.9
N1—C9—C8	122.49 (12)	C20—C21—C22	119.83 (13)
N1—C9—C11	117.30 (12)	C20—C21—H21	120.1
C8—C9—C11	120.14 (12)	C22—C21—H21	120.1
C7—C10—H10A	109.5	C23—C22—C17	117.73 (13)
C7—C10—H10B	109.5	C23—C22—C21	122.75 (13)
H10A—C10—H10B	109.5	C17—C22—C21	119.52 (13)
C7—C10—H10C	109.5	C15—C23—C22	121.13 (12)
H10A—C10—H10C	109.5	C15—C23—H23	119.4
H10B—C10—H10C	109.5	C22—C23—H23	119.4
C9—C11—H11A	109.5	C18—C24—H24A	109.5
C9—C11—H11B	109.5	C18—C24—H24B	109.5
H11A—C11—H11B	109.5	H24A—C24—H24B	109.5
C9—C11—H11C	109.5	C18—C24—H24C	109.5
H11A—C11—H11C	109.5	H24A—C24—H24C	109.5
H11B—C11—H11C	109.5	H24B—C24—H24C	109.5
O1—C12—C13	123.24 (12)		
			
C9—N1—C1—C6	−0.82 (19)	O1—C12—C13—C14	16.4 (2)
C9—N1—C1—C2	179.35 (12)	C8—C12—C13—C14	−164.32 (13)
N1—C1—C2—C3	−178.51 (12)	C12—C13—C14—C15	−177.18 (12)
C6—C1—C2—C3	1.7 (2)	C13—C14—C15—C23	20.6 (2)
C1—C2—C3—C4	0.1 (2)	C13—C14—C15—C16	−161.14 (13)
C2—C3—C4—C5	−1.3 (2)	C17—N2—C16—C15	0.2 (2)
C3—C4—C5—C6	0.6 (2)	C17—N2—C16—Cl1	179.09 (9)
C4—C5—C6—C1	1.2 (2)	C23—C15—C16—N2	0.5 (2)
C4—C5—C6—C7	−179.81 (13)	C14—C15—C16—N2	−177.91 (12)
N1—C1—C6—C5	177.89 (12)	C23—C15—C16—Cl1	−178.36 (9)
C2—C1—C6—C5	−2.28 (19)	C14—C15—C16—Cl1	3.26 (17)
N1—C1—C6—C7	−1.16 (19)	C16—N2—C17—C22	−0.64 (19)
C2—C1—C6—C7	178.67 (12)	C16—N2—C17—C18	179.55 (11)
C5—C6—C7—C8	−176.23 (12)	N2—C17—C18—C19	−179.07 (12)
C1—C6—C7—C8	2.78 (18)	C22—C17—C18—C19	1.12 (19)
C5—C6—C7—C10	2.20 (19)	N2—C17—C18—C24	1.80 (19)
C1—C6—C7—C10	−178.79 (12)	C22—C17—C18—C24	−178.01 (12)
C6—C7—C8—C9	−2.55 (19)	C17—C18—C19—C20	−0.5 (2)
C10—C7—C8—C9	179.08 (12)	C24—C18—C19—C20	178.63 (13)
C6—C7—C8—C12	176.23 (11)	C18—C19—C20—C21	−0.4 (2)
C10—C7—C8—C12	−2.1 (2)	C19—C20—C21—C22	0.5 (2)
C1—N1—C9—C8	1.12 (19)	N2—C17—C22—C23	0.34 (19)
C1—N1—C9—C11	178.05 (11)	C18—C17—C22—C23	−179.86 (11)
C7—C8—C9—N1	0.6 (2)	N2—C17—C22—C21	179.24 (11)
C12—C8—C9—N1	−178.20 (12)	C18—C17—C22—C21	−0.95 (19)
C7—C8—C9—C11	−176.24 (12)	C20—C21—C22—C23	178.96 (12)
C12—C8—C9—C11	4.96 (18)	C20—C21—C22—C17	0.12 (19)
C7—C8—C12—O1	−109.66 (16)	C16—C15—C23—C22	−0.77 (18)
C9—C8—C12—O1	69.14 (18)	C14—C15—C23—C22	177.61 (12)
C7—C8—C12—C13	71.01 (17)	C17—C22—C23—C15	0.40 (19)
C9—C8—C12—C13	−110.19 (14)	C21—C22—C23—C15	−178.47 (12)

### Hydrogen-bond geometry (Å, º)

**Table:**

*D*—H···*A*	*D*—H	H···*A*	*D*···*A*	*D*—H···*A*
C20—H20···O1^i^	0.95	2.58	3.364 (2)	140

Symmetry code: (i) *x*−1, *y*+1, *z*.
